# The Associations between Dairy Product Consumption and Biomarkers of Inflammation, Adipocytokines, and Oxidative Stress in Children: A Cross-Sectional Study

**DOI:** 10.3390/nu12103055

**Published:** 2020-10-06

**Authors:** Hajara Aslam, Felice N Jacka, Wolfgang Marx, Kalliopi Karatzi, Christina Mavrogianni, Eva Karaglani, Mohammadreza Mohebbi, Julie A Pasco, Adrienne O’Neil, Michael Berk, Tzortzis Nomikos, Spyridon Kanellakis, Odysseas Androutsos, Yannis Manios, George Moschonis

**Affiliations:** 1 IMPACT—The Institute for Mental and Physical Health and Clinical Translation, School of Medicine, Barwon Health, Deakin University, Geelong, VIC 3220, Australia; habdussa@deakin.edu.au (H.A.); f.jacka@deakin.edu.au (F.N.J.); wolf.marx@deakin.edu.au (W.M.); julie.pasco@deakin.edu.au (J.A.P.); adrienne.oneil@deakin.edu.au (A.O.); michael.berk@deakin.edu.au (M.B.); 2 Centre for Adolescent Health, Murdoch Children’s Research Institute, Parkville, VIC 3052, Australia; 3 Black Dog Institute, Sydney, NSW 2031, Australia; 4 Biomedical Sciences, James Cook University, Douglas, QLD 4811, Australia; 5 Department of Nutrition and Dietetics, School of Health Science and Education, Harokopio University, 176 76 Athens, Greece; pkaratzi@hua.gr (K.K.); cmavrog@hua.gr (C.M.); ekaragl@hua.gr (E.K.); tnomikos@hua.gr (T.N.); kanellakis@hua.gr (S.K.); 6 Biostatistics Unit, Faculty of Health, Deakin University, Burwood, VIC 3125, Australia; m.mohebbi@deakin.edu.au; 7 Department of Medicine–Western Health, The University of Melbourne, St Albans, VIC 3010, Australia; 8 Department of Epidemiology and Preventive Medicine, Monash University, Prahran, VIC 3800, Australia; 9 Melbourne School of Public Health, University of Melbourne, Melbourne, VIC 3010, Australia; 10 Orygen, The National Centre of Excellence in Youth Mental Health, Centre for Youth Mental Health, Florey Institute for Neuroscience and Mental Health and the Department of Psychiatry, The University of Melbourne, Melbourne, VIC 3010, Australia; 11 Department of Nutrition and Dietetics, School of Physical Education, Sport Science and Dietetics, University of Thessaly, 382 21 Thessaly, Greece; oandroutsos@uth.gr; 12 Department of Dietetics, Nutrition and Sport, School of Allied Health, Human Services and Sport, La Trobe University, Melbourne, VIC 3086, Australia

**Keywords:** milk, dairy products, oxidative stress, inflammation, obesity, leptin, children

## Abstract

The association between dairy product consumption and biomarkers of inflammation, adipocytokines, and oxidative stress is poorly studied in children. Therefore, these associations were examined in a representative subsample of 1338 schoolchildren with a mean age of 11.5 (±0.7) years in the Healthy Growth Study. Information on dairy product consumption was collected by dietary recalls. Total dairy consumption was calculated by summing the intake of milk, yogurt, and cheese. Inflammatory markers, i.e., high-sensitivity C-reactive protein (hs-CRP), interleukin-6 (IL-6), and adipocytokines, i.e., leptin, adiponectin, and the antioxidant enzyme glutathione peroxidase (GPx) were analysed. Due to the skewed distribution hs-CRP, IL-6, and leptin were log transformed. Multivariable regression analyses adjusted for age, sex, energy intake, physical activity, parental education, Tanner stage, and fat mass were used to assess the associations between consumption of total dairy, milk, yogurt, cheese, and markers of inflammation, adipocytokines, oxidative stress, and adiponectin−leptin ratio. Our results showed that milk consumption was inversely associated with leptin (β: −0.101; 95% CI: −0.177, −0.025, *p* = 0.009) and positively associated with the adiponectin−leptin ratio (β: 0.116; 95% CI: 0.020, 0.211; *p* = 0.018), while total dairy, cheese, and yogurt consumption were not associated with inflammatory, adipocytokine, or antioxidant markers. Further prospective studies are needed to confirm these results.

## 1. Introduction

Persistent sub-clinical inflammation is one of the underlying factors that contribute to the pathophysiology of chronic diseases (e.g., cardiovascular disease, type 2 diabetes, depression, and obesity) [1,2] and is characterised by changes in biological markers: an increased concentration of proinflammatory molecules (e.g., C-reactive protein (CRP), interleukin-6 (IL-6), and tumour necrosis factor-α (TNF-α)) and a reduced concentration of anti-inflammatory molecules (e.g., adiponectin) in circulation. Oxidative stress, a phenomenon where the cellular redox balance is compromised due to the over production of reactive oxygen species or reduced compensatory mechanisms, often co-occurs with inflammation and is also correlated with the same chronic conditions [3]. Environmental exposures, particularly diet, may influence disease pathophysiology in part via modulating inflammatory and oxidative stress pathways [4,5,6,7,8,9].

A large body of extant evidence indicates a relationship between dairy product consumption and inflammation in adults. The ATTICA study reported an inverse association between low-fat dairy product consumption and markers related to cardiovascular health (e.g., IL-6, CRP, and TNF-α) in healthy adults [4]. Another cross-sectional study conducted in a representative sample of Brazilian adults demonstrated that yogurt intake was inversely associated with inflammatory markers and cheese intake was positively associated with proinflammatory status, although total dairy and milk intake showed no associations [5]. In a randomised controlled trial, Labonte et al. [6] reported that dairy products did not impart adverse effects on inflammatory markers. Moreover, systematic reviews have indicated that dairy product consumption may be anti-inflammatory and does not exert adverse effects on inflammatory biomarkers [7,10]. A recently published meta-analysis reported positive impacts of dairy consumption on improving inflammatory biomarker profiles in adults [11]. In addition, preliminary evidence also suggests that dairy product consumption may alter cellular redox balance, and this is attributed to some components of dairy such as A1 beta-casein and D-galactose [8,9,12].

Although the role of dairy in modulating inflammation and oxidative stress has been examined in adults, it is yet unclear how dairy product consumption may be related to or modulate such factors in children. However, some evidence demonstrates that dairy intake is inversely associated with conditions such as obesity and cardiometabolic risk factors in children and adolescents, where sub-clinical inflammation is involved in disease prediction and progression [13,14,15]. Therefore, further understanding is warranted on how dairy intake may relate to inflammation and oxidative stress in children. Thus, in a cross-sectional setting, we sought to examine the associations between consumption of total dairy products, milk, yogurt, cheese, and inflammatory markers (i.e., high-sensitivity-CRP (hs-CRP), IL-6), adipocytokines (i.e., leptin and adiponectin), and the antioxidant enzyme glutathione peroxidase (GPx) in children. A representative sample of Greek schoolchildren was utilised for this study [15]. Additionally, we conducted an exploratory analysis to assess the associations between the adiponectin-leptin ratio and dairy product consumption including total dairy, milk, yogurt, and cheese.

## 2. Materials and Methods

### 2.1. Study Population

The Healthy Growth Study (HGS) was a large-scale cross-sectional epidemiological study initiated in May 2007 and completed in June 2009. Approval to conduct the study was granted by the Greek Ministry of National Education and the Ethical Committee of Harokopio University of Athens (16/19.12.2006). The study population was representative of the 9–13 year-old school children living in the four counties under study, which are scattered throughout Greek territory, covering the northern (i.e., Thessaloniki), central (i.e., Attica), western (i.e., Aitoloakarnania), and southern (i.e., Iraklio-Crete) parts of Greece (indicating potential representativeness at a national level). The sampling of schools participating in the HGS was random, multistage, and stratified by parents’ educational level and total population of students attending schools within municipalities of these counties. A detailed letter explaining the aims of the study and a consent form for conducting full measurements was provided to all parents or guardians (“parents” hereinafter) with a child in these schools. Parents who responded positively were asked to sign the consent form and provide their contact details. Data from children and their parents were collected by face-to-face interviews and clinical assessments conducted at school sites. Of the 4145 children who were eligible to participate, 2656 were enrolled in the study (64.1% response) after the parents signed consent on behalf of the children. Detailed methodology has been published elsewhere [16]. Of these 2656 children, a subsample of 1338 children who had full data on both the independent and dependent variable were used for the analysis. The analysis plan for this study was registered at Open Science Framework.

### 2.2. Dietary Intake

A 24-h dietary recall was used to assess the diet of children, including dairy products by trained dietitians and nutritionists. This was done for two consecutive weekdays and one weekend day (i.e., Sunday) in the morning at the school sites. Study participants were asked to describe the type and amount of food, as well as all beverages consumed during the previous day. Food models and sample household measurements (such as cups and spoons) were used to specify serving sizes. In addition, participants were asked about dietary supplements. Further information on packaged and fortified food was also assessed. Food intake data were analysed with Nutritionist V diet analysis software (version 2.1, 1999, First Databank, San Bruno, CA, USA), which was extensively amended to include traditional Greek foods and recipes, as described in the Food Composition Tables and Composition of Greek Cooked Food and Dishes. Information on milk consumption was recorded in mL/d, and cheese and yogurt intake were recorded as g/d (the source for the assessed dairy products were primarily from cow’s milk). The average intake of milk, yogurt, and cheese over three 24-h dietary recalls was considered for the analysis. These values were converted to serves consumed per day according to the Greek dietary guidelines (1 serving of milk = 250 mL; 1 serving of yogurt = 200 g; 1 serving of cheese = 45 g) [17]. Total dairy servings consumed per day were calculated by summing the servings of milk, cheese, and yogurt consumed per day.

### 2.3. Biological Markers

After a 12 h overnight fast, blood samples were obtained for biochemical tests between 8.30–10.30 the following morning. In order to ensure compliance with fasting, reminders were sent to both parents and children on the previous day. A phlebotomist performed venepuncture to obtain a maximum of 25 mL blood. Blood was collected in test tubes with or without ethylenediaminetetraacetic acid (EDTA) as an anticoagulant. Some of the collected blood with and without anticoagulant was centrifuged at 3000 rpm for 15 min to isolate plasma and serum, respectively. The collected plasma and serum samples were pipetted into aliquots of 0.5 mL and were stored at −80 °C. The serum was used to measure the concentration of biological markers, i.e., inflammatory markers (hs-CRP, IL-6), adipocytokines (leptin, adiponectin), and an antioxidant enzyme (GPx). An enzyme-linked immunosorbent assay (ELISA) (R and D Systems, Minneapolis, MN, USA) was used to measure hs-CRP and IL-6 and reported in nmol/L and pg/mL, respectively. Serum leptin levels were measured by a human leptin ELISA, Clinical Range kit (BioVendor Research and Diagnostic products, Karasek, Czech Republic) and was reported in ng/mL. Adiponectin was measured by a Human Adiponectin/Acrp30 Duo Set ELISA kit (R&D Systems, Minneapolis, MN, USA) and reported in μg/mL. From this information, the adiponectin−leptin ratio was calculated (marker of adipocyte dysfunction). Serum GPx activity was determined according to the recycling method of Wendel [18]. The oxidized glutathione (GSSG) formed during GPx reaction with H_2_O_2_ and glutathione (GSH) was reduced by an excess of glutathione reductase activity providing a constant level of GSH. The concomitant oxidation of nicotinamide adenine dinucleotide phosphate (NADPH) was monitored photometrically by the decrease of absorbance at 340 nm for 5 min at 25 °C using a microplate reader (PowerWave XS, BioTek, Winooski, VT, USA). The activity was expressed as Units/mL.

### 2.4. Anthropometrics and Physical Measures

A digital scale (Seca Alpha, Model 770, Hamburg, Germany) was used to measure body weight to the nearest 10 g in the minimum clothing possible. A commercial stadiometer (Leicester Height Measure, Invicta Plastics Ltd., Oadby, UK) was used to measure the height to the nearest 0.1 cm with participants barefoot, shoulders in a relaxed position, arms hanging freely, and head aligned in the Frankfort plane. Weight and height were converted to body mass index (BMI) using Quetelet’s equation (weight (kg)/height^2^ (m^2^)). Bioelectrical impedance analysis (Akkern BIA 101; Akkern Srl., Florence, Italy) was used to measure fat mass (FM) of participants. Participants were advised to refrain from any food intake (i.e., solid or liquid) and strenuous exercise for 4 h prior to measurement; additionally, participants were instructed to avoid wearing any metal objects during the measurement. The same protocol and equipment were used to collect anthropometric and body composition measures across all schools. A paediatrician determined the pubertal stage of girls and boys by visually inspecting the breast and genital characteristics, respectively. Based on the pubertal stage children were classified into five Tanner stages [19]. The earliest stage of puberty was indexed by Tanner stage 1.

### 2.5. Sociodemographic Factors

Parental educational level (years of education, e.g., <9 yrs, 9–12 yrs, >12 yrs) was used as the surrogate measure for socio-economic status. This information was obtained from parents (predominantly mothers) during the scheduled face-to-face interviews at school or during a telephone interview for parents unable to attend on-site face-to-face interviews (approximately 5% of the total sample).

### 2.6. Physical Activity Levels

Physical activity was measured by step counters. Children were provided with and instructed to wear a waist-mounted pedometer (Yamax SW-200 Digiwalker, Tokyo, Japan) for one week, i.e., from Monday to Sunday, and record the number of steps on a daily basis that appeared on the pedometer screen [20].

### 2.7. Statistical Analysis

Characteristics of children were described by mean (± SD), median (interquartile range (IQR)), or relative frequencies (%) stratified by total dairy consumption categories (<1 serving/d, 1–<3 servings/d, 3–4 servings/d, >4 servings/d). One-way ANOVA or Kruskal–Wallis-H test for continuous data and Chi-square test (or Fisher’s exact test) for categorical data were used to compare characteristics across total dairy consumption categories. A post-hoc pairwise comparison was conducted when the null hypothesis was rejected (*p* < 0.05). Prior to analysis hs-CRP, IL-6, and leptin were log transformed due to their skewed distribution. First, univariate regression analysis models were performed to obtain the unadjusted beta-coefficients and 95% confidence interval (CI) between consumption of total dairy (as a continuous and categorical variable), milk, yogurt, cheese, and biological markers (i.e., hs-CRP, IL-6, leptin, adiponectin, GPx). Covariates such as age, sex, total number of steps, parental education (i.e., mother’s and father’s education), dietary fibre, fat, carbohydrate intake, Tanner stage, and energy intake were sequentially tested in bivariate models to assess their relationship with both the independent and dependent variable. Covariates that were significantly related to or changed the beta-coefficient, when added or removed from the model qualified for the final multivariable models. Age, sex, total number of steps, parental education, Tanner stage, and energy intake qualified as covariates for the multivariable model (model 1). As FM is a strong predictor of the assessed biological markers [21], FM was included as a covariate in a separate model (model 2) in addition to the covariates considered in model 1. In all models, potential interactions were tested and partial eta-squared (effect size) was calculated to determine the strength of the association between the independent and dependent variables. Additionally, the association between consumption of total dairy, milk, yogurt, cheese, and the adiponectin–leptin ratio were assessed in separate models as an exploratory analysis. For all analyses, StataIC 16 was used.

Children who had data on both the independent (total dairy consumption) and dependent (inflammation and oxidative stress markers) variables were included in the analysis (*n* = 1338). Two children had missing information on FM and there were no missing data for other variables.

## 3. Results

Table 1 presents the characteristics of children according to their total dairy consumption categories. Of the 1338 children, 15.5% (*n* = 208) consumed the recommended servings of total dairy per day (3–4 servings/d) according to the Greek dietary guidelines. Nearly 75.3% of children consumed below (i.e., <1 serving/d or 1–<3 servings/d) the recommended servings of total dairy per day, while only 9.2% children consumed above the recommended servings of total dairy per day. Children who consumed 3–4 servings/d of total dairy were younger than children who consumed <1 serving/d (*p* = 0.028). The intake of macronutrients (i.e., protein, fat, dietary fibre, and carbohydrate intake) including total energy were higher in children who consumed >4 servings/d of total dairy compared to those consuming <1 serving/d (*p* = <0.001). Additionally, children who consumed >4 servings/d of total dairy had lower BMI (*p* = 0.009) compared to those consuming <1 serving/d. Children who consumed the recommended servings of total dairy (*n* = 208) reported the highest number of steps per day compared to other categories of dairy consumption. There were no differences detected for other variables across the dairy consumption categories (Table 1).

Total dairy intake was considered as both a categorical and continuous variable (in separate models) when assessing the association with biological markers. When total dairy consumption was considered as a categorical variable, the univariate analysis showed that consumption of >4 servings/d total dairy was associated with reduced level of log leptin compared to consumption of 1 serving/d of total dairy (β: −0.379; 95% CI: −0.602, −0.157; *p* = 0.001). Similarly, when total dairy consumption was considered as a continuous variable, the univariate analysis demonstrated an inverse relationship between total dairy and log leptin (β: −0.061; 95%CI: −0.102, −0.020; *p* = 0.004). However, after adjusting for covariates (i.e., model 1 and model 2) the associations between total dairy intake (both as continuous and categorical variables) and log leptin were no longer significant (Table 2). Furthermore, there was no association between total dairy (both as continuous and categorical variables) and other biological markers (i.e., hs-CRP, IL-6 and adiponectin, GPx) in either the adjusted (i.e., model 1 and model 2) or unadjusted models (Table 2).

**Figure 1:**
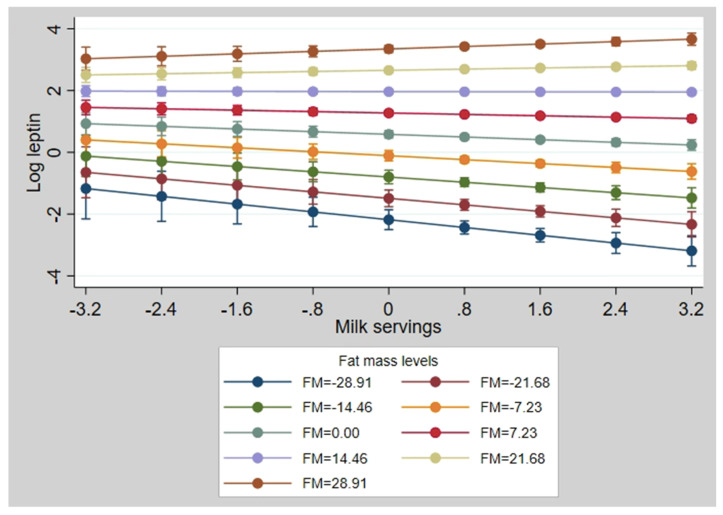
Interaction plot for log leptin at different fat mass (FM) levels. The values for milk servings, log leptin, and FM are presented in z-scores.

The univariate association between milk consumption and log leptin showed a significant inverse association (β: −0.148; 95% CI: −0.211, −0.086; *p* = 0.0001). Adjustment for covariates in model 1 did not change the significance of the association (β: −0.082; 95% CI: −0.144, −0.020; *p* = 0.010). In model 2, which was further adjusted for FM, a significant inverse association between milk and log leptin (β: −0.101; 95% CI: −0.177, −0.025; *p* = 0.009) was demonstrated, but this association was dependent on the FM level (Figure 1). Milk consumption was not associated with other biological markers (i.e., hs-CRP, IL-6, adiponectin, GPx) (Table 3).

The results of either the univariate or multivariable analysis did not show an association between yogurt consumption and any of the biological markers (i.e., hs-CRP, IL-6, leptin, adiponectin, GPx). When cheese consumption was considered, the univariate analysis indicated no association between cheese consumption and any of the biological markers. However, cheese consumption showed a positive association with log hs-CRP (β: 0.109; 95% CI: 0.017, 0.201; *p* = 0.020) and log leptin (β: 0.091; 95% CI: 0.028, 0.154; *p* = 0.005) in model 1. In model 2, which was further adjusted for FM, cheese consumption was not significantly associated with either log hs-CRP or log leptin. Additionally, in model 2 no associations were observed between cheese consumption and other biological markers (i.e., IL-6, adiponectin, GPx).

The association between the adiponectin−leptin ratio and dairy product intake is shown in Table 4. The univariate association between milk consumption and the adiponectin−leptin ratio showed a significant positive association (β: 0.159; 95% CI: 0.086, 0.231; *p* = 0.0001). Adjustment for covariates in model 1 did not change the significance of this association (β: 0.089; 95% CI: 0.017, 0.162; *p* = 0.016). In model 2, a positive association between milk and the adiponectin−leptin ratio (β: −0.116; 95% CI: 0.020, 0.0211; *p* = 0.018) was demonstrated, and this association was dependent on the FM level. Cheese consumption was inversely associated with the adiponectin−leptin ratio only in model 1 (β: −0.078; 95% CI: −0.152, −0.004; *p* = 0.039).

## 4. Discussion

In this cross-sectional study conducted with a representative sample of Greek school-aged children, we found evidence that milk consumption was inversely associated with leptin levels; however, this association differed according to FM levels and was weak. A positive association was found between cheese intake and leptin and hs-CRP levels in the model adjusted for age, sex, energy intake, total number of steps, parental education, and Tanner stage; however, further adjustment for FM attenuated the association, suggesting that this relationship is fully explained by FM. No association was found between total dairy intake, milk, or yogurt intakes and hs-CRP, IL-6, adiponectin, or GPx.

Leptin is one of the most abundant adipocytokines produced by adipocytes together with cytokines such as tumour necrosis-α, IL-6, IL-1, CC-chemokine ligand 2, and other mediators [22]. Moreover, the adipocytes produce leptin in proportion to the body fat stores predisposing obese individuals to have increased levels of leptin in circulation [23,24,25]. In our study, we found an inverse association between milk and leptin levels, and this association was sustained even after adjusting for confounders such as age, sex, energy intake, total number of steps, parental education, and Tanner stage. Further adjustment for FM with an interaction term with milk retained the inverse significant association between milk and leptin, indicating that this association differed according to FM levels. Indeed, at lower FM levels the inverse association between milk intake and leptin was more prominent. As higher body fat is proportional to higher serum leptin levels, previous findings demonstrating inverse associations between milk intake and obesity in children may partly be explained in light of our findings [14,26]. Additionally, in the exploratory analysis, we found that milk intake was positively associated with the adiponectin–leptin ratio. A lower adiponectin–leptin ratio is considered a predictive marker of adipose tissue dysfunction, chronic low-grade inflammation, and metabolic syndrome [27,28]. Therefore, our finding suggests that milk intake may have some beneficial properties in terms of alleviating the aforementioned conditions in children of this age group.

Moreover, it is increasingly understood that leptin possesses pro-inflammatory properties [22,29,30]. Leptin may upregulate the production of inflammatory cytokines such as CRP, TNF-α, IL-6, and IL-12 and be involved in low-grade inflammation, which plays a central role in the pathophysiology of obesity, depression, metabolic and cardiovascular disease [2,22,31,32,33]. The inverse association between serum leptin and milk consumption demonstrated by the present results also suggests that milk may have anti-inflammatory properties and is concordant with previous findings [4,7]. Although the mechanistic pathways by which milk might alleviate inflammation is not completely understood, milk contains nutrients such as calcium, vitamin D, bio active peptides, and linoleic acid, which possess anti-inflammatory properties and act in distinct pathways to mitigate inflammation [34,35,36]. However, recent evidence indicates that the impact of dairy on health outcomes cannot be attributed to a single element present in dairy, and the overall nutrient composition or so-called the matrix effect needs to be considered [37]. Nevertheless, we did not find an association between milk intakes and other inflammatory markers i.e., hs-CRP, IL-6, or adiponectin in children.

Regarding fermented dairy intake (i.e., cheese and yogurt), we did not detect any association between cheese, yogurt intake, and markers of inflammation. Our findings are discordant with the results of previous studies in adults, which suggest anti-inflammatory properties for yogurt and conversely, pro-inflammatory properties for cheese [5,7]. Gonzalez-Gill et al. [38] studied the cross-sectional relationship between 41 food items including dairy products and hs-CRP in children and showed that vegetable consumption was strongly related to reduced levels of hs-CRP [38]. However, strong evidence was not found to show that any of the dairy products (i.e., milk, yogurt, and cheese) were related to hs-CRP. Similarly, in our study yogurt, cheese, or milk consumption were not associated with hs-CRP; however, we did assess a range of other biomarkers and their relationships with yogurt and cheese consumption and found no associations. We also assessed the associations between total dairy, milk, yogurt, cheese, and the antioxidant enzyme marker GPx. Glutathione and GPx are molecules that are involved in cellular redox balance with the main task of keeping free radicals in check. Milk and dairy products are sources of precursor molecules such as cysteine and selenium that are needed for the proper functioning of these redox molecules. In this regard, intakes of dairy products may aid in positively regulating cellular redox balance [39,40,41,42,43]. Conversely, some evidence has indicated that certain milk protein types (i.e., A1-beta casein) and sugars (i.e., D-galactose) can negatively alter the cellular redox balance that may have detrimental implications to health [8,9,12]. However, our study detected no positive or negative associations between consumption of dairy products including milk, yogurt, or cheese, and the antioxidant enzyme marker GPx.

A strength of the HGS is the use of a representative population-based sample of Greek schoolchildren due to its random, multistage, stratified sampling procedure. For the first time in this study, we assessed the association between dairy consumption with reference to Greek dietary guidelines and biological markers of inflammation, adipocytokines, and oxidative stress; this was to investigate whether consuming the recommended servings of dairy per day had any association with biological markers in addition to the other expected health benefits of dairy consumption such as bone health. However, we did not find evidence to suggest that consumption of the recommend servings of total dairy per day had any positive impacts on biological markers compared to the under- and over-consumption of dairy products. As dairy products differ in their biological profile, we also analysed the individual correlations of milk, yogurt, cheese, and biological markers in separate models. In addition, the sample size was among the strengths of this study. A post-hoc power calculation based on available sample size showed that there was more than 90% statistical power to detect small effect sizes in the multivariable regression models.

Our study had some limitations. Firstly, although rigorously trained dietitians were involved in collection of dietary data from participants, recall and reporting bias could not be completely avoided. Furthermore, when calculating the children’s total dairy intake, we only considered the predominant forms of dairy that were recommended by the Greek dietary guidelines, i.e., milk (whole, skim, low fat), yogurt (full fat, low fat, 10% fat), and cheese (white, yellow) and did not account for other dairy forms such as ice-cream and cream. Importantly, given the number of tests we conducted, it is also possible that our significant findings represent Type 1 (false positive) errors. Lastly, as this was a cross-sectional study no causality can be attributed, and further longitudinal or randomised controlled trials are warranted to confirm our results.

## 5. Conclusions

In conclusion, our study results revealed that milk consumption is weakly associated with lower leptin concentrations in children and positively associated with the adiponectin–leptin ratio, suggesting potential cardioprotective protective properties of milk consumption in children of this age group. However, we did not detect any association between total dairy, cheese, or yogurt intake and adipocytokines in children. Although dairy products differ in terms of biological and nutritional profiles, and these variations may differentially impact inflammatory markers, adipocytokines, and redox markers, the present data did not provide evidence for differential associations between various dairy types and inflammatory or oxidative stress markers in children. Further research is needed to investigate these associations in greater depth.

## Figures and Tables

**Table 1:** Descriptive characteristics of study participants by categories of dairy products consumption.

Variables	<1 Serving/d	1–<3 Servings/d	3–4 Servings/d	>4 Servings/d	*p*-Value
	*n* = 170	*n* = 837	*n* = 208	*n* = 123	
Age (yrs)	** 11.3 (0.7)	11.1 (0.7)	** 11.1 (0.6)	11.2 (0.6)	0.007
Sex, n (%)					0.0001
Females	108 (63.5)	436 (52.1)	102 (49.0)	43 (35.0)	
**Dietary indices**					
Total energy (Kcal/d)	** 1555.9 (528.9)	1723.6 (499.7)	1968.2 (477.8)	** 2375.0(690.9)	0.0001
Protein (g/d)	** 54.9 (22.2)	65.9 (21.4)	78.6 (19.1)	** 99.8 (28.4)	0.0001
Fat (g/d)	** 68.5 (27.8)	78.0 (27.4)	92.0 (26.0)	** 113.2 (38.4)	0.0001
Dietary fibre (g/d)	** 10.6 (8.1–14.5)	11.9 (8.5–16.6)	12.8 (9.7–17.8)	** 14.3 (11.2–20.4)	0.0001
Carbohydrates (g/d)	** 189.5 (77.4)	198.9 (67.0)	216.6 (68.4)	** 250.4 (90.8)	0.0001
**Dairy intake**					
Milk (servings/d)	** 0.0 (0.0–0.49)	1.0 (0.8–1.5)	1.9 (1.5–2.1)	** 2.0 (1.6–2.9)	0.0001
Yogurt (servings/d)	** 0.0 (0.0–0.0)	0.0 (0.0–0.0)	0.0 (0.0–0.3)	** 0.0 (0.0–0.1)	0.0001
Cheese (servings/d)	** 0.3 (0.0–0.6)	0.7 (0.3–1.1)	1.4 (1.0–1.8)	** 2.4 (1.8–3.2)	0.0001
**Inflammatory and oxidative stress indices**					
hs-CRP (ng/mL)	451.0 (156.0–1403)	479.0 (187.0–1415.0)	556.0 (234.5–1369.0)	418.0 (181.0–939)	0.407
IL-6 (pg/mL)	0.9 (0.6–1.2)	0.8 (0.6–1.2)	0.9 (0.6–1.2)	0.7 (0.5–1.1)	0.312
Leptin (ng/mL)	** 8.7 (3.4–17.1)	7.1 (3.7–14.0)	7.1 (3.5–12.8)	** 4.7 (2.6–9.5)	0.0002
Adiponectin (μg/mL)	6.9 (3.7)	7.0 (3.1)	6.6 (3.3)	7.3 (3.4)	0.242
GPx activity (U/mL)	0.057 (0.013)	0.057 (0.014)	0.057 (0.014)	0.056 (0.016)	0.850
**Anthropometric and physical indices**					
Height (cm)	149.1 (8.3)	148.7 (7.7)	148.8 (8.0)	148.3 (7.2)	0.855
BMI (kg/m2)	** 20.9 (4.1)	20.2 (3.5)	20.4 (4.0)	** 19.6 (3.6)	0.014
Fat Mass (kg)	13.4 (9.2–20.1)	12.6 (8.6–18.1)	12.4 (8.2–18.3)	10.7 (6.9–15.7)	0.015
Weight status, n (%)					0.01
Underweight	5 (2.9)	17 (2.0)	6 (2.9)	11 (8.9)	
Normal weight	85 (50.0)	468 (55.9)	119 (57.2)	71 (57.7)	
Overweight	55 (32.4)	271 (32.4)	57 (27.4)	26 (21.2)	
Obese	25 (14.7)	81 (9.7)	26 (12.5)	15 (12.2)	
**Tanner stage, *n* (%)**					0.065
Stage 1	45 (26.5)	269 (32.2)	52 (25.0)	42 (34.1)	
Stage 2	68 (40.0)	346 (41.3)	99 (47.6)	52 (42.3)	
Stage 3	37 (21.8)	165 (19.7)	42 (20.2)	20 (16.3)	
Stage 4	12 (7.0)	47 (5.6)	13 (6.2)	8 (6.5)	
Stage 5	8 (4.7)	10 (1.2)	2 (1.0)	1 (0.8)	
**Physical activity indices**					
Average total steps per day	** 11,832.0 (4398.2)	** 13,343.0 (5241.6)	** 13,685.8 (4905.4)	** 13,521.5 (4106.8)	0.0012
**Socio economic status**					
Father’s education, *n* (%)					0.033
<9 yrs	48 (28.2)	198 (23.7)	35 (16.8)	28 (22.8)	
9–12 yrs	72 (42.4)	298 (35.6)	79 (38.0)	46 (37.4)	
>12 yrs	50 (29.4)	341 (40.7)	94 (45.2)	49 (39.8)	
Mother’s education, *n* (%)					0.080
<9 yrs	43 (25.3)	155 (18.5)	35 (16.8)	20 (16.3)	
9–12 yrs	73 (42.9)	314 (37.5)	79 (38.0)	50 (40.6)	
>12 yrs	54 (31.8)	368 (44.0)	94 (45.2)	53 (43.1)	

Data are shown as mean (±SD), median (interquartile range (IQR)) or n (%); Missing variables: fat mass (n = 2); ** Bonferroni corrected *p* values (*p* < 0.05, *p* < 0.001); high sensitivity C-reactive protein (hs-CRP); interleukin 6 (IL-6); glutathione peroxidase (GPx).

**Table 2:** Associations between total dairy consumption and markers of inflammation, adipocytokines, and oxidative stress.

Independent Variable(s)	Dependent Variable(s)									
	hs-CRP		IL-6		Leptin		Adiponectin		GPx	
	β-Coefficient and 95% CI	Partial Eta-Squared	β-Coefficient and 95% CI	Partial Eta-Squared	β-Coefficient and 95% CI	Partial Eta-Squared	β-Coefficient and 95% CI	Partial Eta-Squared	β-Coefficient and 95% CI	Partial Eta-Squared
**Total dairy (categorical)**										
Unadjusted model										
<1 serving/d	Reference	0.002	Reference	0.002	Reference	0.010	Reference	0.003	Reference	0.0006
1–<3 servings/d	0.093 (−0.128, 0.313)		0.008 (−0.097, 0.112)		−0.068 (−0.226, 0.900)		0.089 (−0.446, 0.624)		−0.0006 (−0.0029, 0.0018)	
3–4 servings/d	0.200 (−0.070, 0.471)		0.013 (−0.115, 0.141)		−0.073 (−0.268, 0.121)		−0.317 (−0.974, 0.341)		0.0001 (−0.0028, 0.0030)	
>4 servings/d	−0.023 (−0.333, 0.287)		−0.089 (−0.236, 0.058)		** −0.379 (−0.602, −0.157)		0.393 (−0.360, 1.145)		−0.0011 (−0.0044, 0.0022)	
Adjusted model 1										
<1 serving/d	Reference	0.003	Reference	0.001	Reference	0.003	Reference	0.004	Reference	0.0023
1–<3 servings/d	0.107 (−0.115, 0.329)		0.020(−0.087, 0.126)		0.070 (−0.082, 0.223)		0.023 (−0.518, 0.603)		−0.0001 (−0.0025, 0.0022)	
3–4 servings/d	0.249 (−0.028, 0.526)		0.027 (−0.196, 0.159)		0.106 (−0.084, 0.296)		−0.258 (−0.939, 0.411)		0.0016 (−0.0014, 0.0045)	
>4 servings/d	0.073 (−0.255, 0.400)		−0.052 (−0.209, 0.105)		−0.066 (−0.292, 0.159)		0.600 (−0.199, 1.440		0.0015 (−0.0020, 0.0050)	
Adjusted model 2										
<1 serving/d	Reference	0.002	Reference	0.0007	Reference	0.003	Reference	0.003	Reference	0.0020
1–<3 servings/d	0.124 (−0.069, 0.318)		0.022 (−0.082, 0.127)		0.090 (−0.005, 0.185)		0.010 (−0.526, 0.547)		−0.0001 (−0.0025, 0.0022)	
3–4 servings/d	0.203 (−0.038, 0.445)		0.018 (−0.113, 0.148)		0.057 (−0.062, 0.176)		−0.226 (−0.895, 0.442)		0.0016 (−0.0014, 0.0045)	
>4 servings/d	0.152 (−0.135, 0.438)		−0.037 (−0.191, 0.117)		0.018 (−0.122, 0.159)		0.545 (−0.247, 1.337)		0.0015 (−0019, 0.0050)	
**Total dairy continuous (servings/d)**										
Unadjusted model	0.025 (−0.033, 0.082)	0.001	−0.017 (−0.044, 0.010)	0.001	** −0.061(−0.102, −0.020)	0.006	0.029 (−0.111, 0.168)	0.0001	−0.0001 (−0.0007, 0.0005)	0.0001
Adjusted model 1	0.052 (−0.010, 0.115)	0.002	−0.010 (−0.040, 0.020)	0.0003	0.001 (−0.042, 0.045)	<0.0001	0.085 (−0.068, 0.238)	0.0009	0.0005 (−0.0002, 0.0012)	0.0017
Adjusted model 2	0.050 (−0.004, 0.105)	0.002	−0.104 (−0.040, 0.019)	0.0004	−0.001 (−0.028, 0.026)	<0.0001	0.086 (−0.065, 0.238)	0.0009	0.0005 (−0.0002, 0.0012)	0.0017

**p < 0.01; Model 1: adjusted for age, sex, energy intake, total number of steps, parental education (i.e., mother’s and father’s education), Tanner stage; Model 2: adjusted for age, sex, energy intake, total number of steps, parental education, Tanner stage, FM; fat mass (FM); high sensitivity C-reactive protein (hs-CRP); interleukin 6 (IL-6); glutathione peroxidase (GPx).

**Table 3:** Associations between milk, yogurt, cheese consumption, and markers of inflammation, adipocytokines, and oxidative stress.

Independent Variable(s)	Dependent Variable(s)									
	hs-CRP		IL-6		Leptin		Adiponectin		GPx	
	β-Coefficient and 95% CI	Partial Eta-Squared	β-Coefficient and 95% CI	Partial Eta-Squared	β-Coefficient and 95% CI	Partial Eta-Squared	β-Coefficient and 95% CI	Partial Eta-Squared	β-Coefficient and 95% CI	Partial Eta-Squared
**Milk (servings/d)**										
Unadjusted model	−0.013 (−0.101, 0.075)	<0.0001	−0.016(−0.058, 0.026)	0.0004	*** −0.148(−0.211, −0.086)	0.016	0.066 (−0.148, 0.279)	0.003	−0.0002 (−0.0011, 0.0007)	0.0001
Adjusted model 1	−0.005 (−0.095, 0.086)	<0.0001	−0.008 (−0.052, 0.035)	0.0001	* −0.082 (−0.144, −0.020)	0.005	0.054 (−0.166, 0.274)	0.0002	0.0002 (−0.0008, 0.0012)	0.0001
Adjusted model 2	0.063 (−0.016, 0.142)	0.002	0.004 (−0.038, 0.047)	<0.0001	** −0.101 (−0.177, −0.025) ^ψ^	0.005	0.006 (−0.213, 0.225)	<0.0001	0.0002 (−0.0007, 0.0012)	0.0002
**Yogurt (servings/d)**										
Unadjusted model	−0.002 (−0.261, 0.257)	<0.0001	−0.067 (−0.190, 0.552)	0.0005	−0.012 (−0.199, 0.174)	<0.0001	−0.052 (−0.681, 0.576)	<0.0001	−0.0009 (−0.0037, 0.0018)	0.0003
Adjusted model 1	0.068 (−0.189, 0.326)	0.0002	−0.056 (−0.179, 0.067)	0.0006	−0.020 (−0.197, 0.157)	<0.0001	−0.056 (−0.622, 0.634)	<0.0001	−0.0008 (−0.0035, 0.0020)	0.0002
Adjusted model 2	0.096 (−0.129, 0.320)	0.0005	−0.050 (−0.171, 0.071)	0.0004	0.011 (−1.000, 0.122)	<0.0001	−0.014 (−0.636, 0.608)	<0.0001	−0.0007 (−0.0035, 0.0020)	0.0002
**Cheese (servings/d)**										
Unadjusted model	0.068 (−0.018, 0.154)	0.0018	−0.015 (−0.056, 0.026)	0.0004	0.007 (−0.055, 0.069)	<0.0001	0.007 (−0.201, 0.216)	<0.0001	0.0001 (−0.0008, 0.0090)	<0.0001
Adjusted model 1	* 0.109 (0.017–0.201)	0.004	−0.006 (−0.050, 0.038)	<0.0001	** 0.091 (0.028, 0.154)	0.006	0.126 (−0.098, 0.351)	0.0009	0.001 (−0.00002, 0.0020)	0.003
Adjusted model 2	0.032 (−0.486, 0.113)	0.0004	−0.021 (−0.064, 0.023)	0.0007	0.007 (−0.033, 0.046)	<0.0001	0.184 (−0.039, 0.407)	0.002	0.001 (−0.00002, 0.0019)	0.003

*** *p* < 0.0001 ** *p* < 0.01 * *p* < 0.05; Model 1: adjusted for age, sex, energy intake, total number of steps, parental education (i.e., mother’s and father’s education), Tanner stage; Model 2: adjusted for age, sex, energy intake, total number of steps, parental education (i.e., mother’s and father’s education) Tanner stage, FM; ψ: resultant coefficient after accounting for the interaction between milk and FM; fat mass (FM); high sensitivity C-reactive protein (hs-CRP); interleukin 6 (IL-6); glutathione peroxidase (GPx).

**Table 4:** Associations between total dairy, milk, yogurt, cheese consumption, and the adiponectin−leptin ratio.

Independent Variable(s)	Dependent Variable: Adiponectin-Leptin Ratio			
	β-Coefficient	95%Confidence Interval	Significance	Partial Eta-Squared
**Total dairy continuous (servings/d)**				
Unadjusted model	0.064	0.016, 0.112	0.009	0.005
Adjusted model 1	0.008	−0.043, 0.058	0.763	<0.0001
Adjusted model 2	0.010	−0.024, 0.044	0.563	0.0003
**Milk (servings/d)**				
Unadjusted model	0.159	0.086, 0.231	0.0001	0.014
Adjusted model 1	0.089	0.017, 0.162	0.016	0.004
Adjusted model 2	0.116 ^ψ^	0.020, 0.211	0.018	0.004
**Yogurt (servings/d)**				
Unadjusted model	−0.001	−0.216, 0.214	0.994	<0.0001
Adjusted model 1	0.013	−0.194, 0.221	0.899	<0.0001
Adjusted model 2	−0.021	−0.160, 0.119	0.771	<0.0001
**Cheese (servings/d)**				
Unadjusted model	−0.009	−0.080, 0.063	0.812	<0.0001
Adjusted model 1	−0.078	−0.152, −0.004	0.039	0.003
Adjusted model 2	0.016	−0.034, 0.066	0.531	0.0003

Model 1: adjusted for age, sex, energy intake, total number of steps, parental education (i.e., mother’s and father’s education), Tanner stage; Model 2: adjusted for age, sex, energy intake, total number of steps, parental education (i.e., mother’s and father’s education), Tanner stage, FM; ψ: resultant coefficient after accounting for the interaction between milk and FM; fat mass (FM).
